# Epitranscriptomic Insights: RNA m5C Effector Proteins as Key Players in Brain Disease

**DOI:** 10.1002/alz70855_099464

**Published:** 2025-12-23

**Authors:** Adriana PerezGrovas‐Saltijeral, Helen Miranda Knight, Enya Murray

**Affiliations:** ^1^ University of Nottingham, Nottingham, Nottingham, United Kingdom

## Abstract

**Background:**

Neurodegenerative diseases like Alzheimer's disease (AD) and prodromal stages such as Mild Cognitive Impairment involve dysregulated RNA processing. RNA methylation, a post‐transcriptional RNA modification, is an important regulator of RNA stability, processing, and transport, and is mediated by RNA‐binding proteins known as writers, readers, and erasers. 5‐methylcytosine (m^5^C) is abundant in brain mRNAs, tRNAs, rRNAs and mt‐RNA, and influences ribosomal activity and translational dynamics.

**Methods:**

We examined if changes in the m^5^C RNA methylation system could contribute to neurodegenerative disorders. Human brain RNA‐seq data from the Aging, Dementia, and Traumatic Brain Injury (TBI) Study were used to compare m^5^C effector protein expression between AD patients, TBI individuals, and age‐matched controls, and correlated with AD neuropathological scores, Braak and CERAD stages. Furthermore, we assessed abundance of NSUN5, a m^5^C rRNA methyltransferase, by using confocal microscopy and brain tissue from individuals with MCI and healthy controls across different cell populations.

**Results:**

Altered expression of m^5^C writers (NSUN6 and NSUN7) and reader (ALYREF) protein transcripts was observed in AD and TBI across different brain regions. NSUN6 expression was found significantly reduced in the superior temporal gyrus (STG) and white matter, while NSUN7 showed increased expression in the hippocampus, correlating with Braak and CERAD stages. TBI tissue also exhibited reduced NSUN6 expression in the STGs, regardless of AD diagnosis. ALYREF showed significant differences in relative expression across Braak stages in the hippocampus and inferior parietal lobe. NSUN5 protein abundance in the human frontal gyrus was higher in pyramidal cells than glial cells overall, and significantly higher in healthy controls compared to MCI tissue.

**Conclusion:**

These findings indicate consistent alterations in transcript expression of the m^5^C RNA methylation machinery in neurocognitive disorders. Our findings are consistent with evidence that mutations of m^5^C effector genes cause neurodevelopmental disorders, directly linking m^5^C and its regulator machinery with neurological processes. Evidence suggests that m^5^C effector proteins might play diverse roles, localizing to compartments such as synaptic processes. As m^5^C modifications regulate synaptic function, mitochondrial metabolism and neuronal health, they are promising novel targets for therapeutic intervention and biomarker development in cognitive diseases.

